# Absolute Eye Gaze Estimation With Biosensors in Hearing Aids

**DOI:** 10.3389/fnins.2019.01294

**Published:** 2019-12-05

**Authors:** Antoine Favre-Félix, Carina Graversen, Tanveer A. Bhuiyan, Martin A. Skoglund, Sergi Rotger-Griful, Mike Lind Rank, Torsten Dau, Thomas Lunner

**Affiliations:** ^1^Eriksholm Research Centre, Snekkersten, Denmark; ^2^Hearing Systems Section, Department of Health Technology, Technical University of Denmark, Lyngby, Denmark; ^3^Division of Automatic Control, Linköping University, Linköping, Sweden; ^4^UNEEG Medical A/S, Lynge, Denmark

**Keywords:** eye gaze estimation, electrooculography, EarEOG, inertial sensors, head tracking, hearing aids, hearing-impaired

## Abstract

People with hearing impairment typically have difficulties following conversations in multi-talker situations. Previous studies have shown that utilizing eye gaze to steer audio through beamformers could be a solution for those situations. Recent studies have shown that in-ear electrodes that capture electrooculography in the ear (EarEOG) can estimate the eye-gaze relative to the head, when the head was fixed. The head movement can be estimated using motion sensors around the ear to create an estimate of the absolute eye-gaze in the room. In this study, an experiment was designed to mimic a multi-talker situation in order to study and model the EarEOG signal when participants attempted to follow a conversation. Eleven hearing impaired participants were presented speech from the DAT speech corpus (Bo Nielsen et al., 2014), with three targets positioned at −30°, 0° and +30° azimuth. The experiment was run in two setups: one where the participants had their head fixed in a chinrest, and the other where they were free to move their head. The participants’ task was to focus their visual attention on an LED-indicated target that changed regularly. A model was developed for the relative eye-gaze estimation, taking saccades, fixations, head movement and drift from the electrode-skin half-cell into account. This model explained 90.5% of the variance of the EarEOG when the head was fixed, and 82.6% when the head was free. The absolute eye-gaze was also estimated utilizing that model. When the head was fixed, the estimation of the absolute eye-gaze was reliable. However, due to hardware issues, the estimation of the absolute eye-gaze when the head was free had a variance that was too large to reliably estimate the attended target. Overall, this study demonstrated the potential of estimating absolute eye-gaze using EarEOG and motion sensors around the ear.

## Introduction

Hearing-impaired (HI) people are commonly challenged in situations with multiple simultaneous sound sources, like interfering speakers in a restaurant or at a social gathering, even if sound amplification is provided through hearing aids (HA) (Arons, 2000; Bee and Micheyl, 2008). To improve the listening situation for the HI people, HA signal processing strategies have been developed, such as directional filtering (beamforming), dynamic range compression, and background noise reduction (Puder, 2009). However, ideally, these algorithms require an estimate of the sound source (target) the listener is attending to in order to be able to enhance the target-to-noise ratio and thus improve the benefit for the user.

Recent advances in electrophysiology suggest that the attended sound source can be estimated via electroencephalography (EEG) or electrooculography (EOG) in combination with head tracking (Mesgarani and Chang, 2012; O’Sullivan et al., 2015; Pomper and Chait, 2017). While it has been shown that attended sound sources can be decoded from cortical brain responses via a correlation analysis of the scalp responses and an envelope representation of the attended input sound stream, a robust estimate requires several seconds of decoding, which is not applicable for real-time steering (Fuglsang et al., 2017; Dau et al., 2018). In contrast, in a proof-of-concept study (Favre-Félix et al., 2018) recently demonstrated the potential of improving speech intelligibility in HI people in real time by assessing visual attention via EOG. Although the results of Favre-Félix et al. (2018) provided first insights into visual attention steering via EOG, the study had substantial limitations regarding its potential value for applicability in HA technology. In that study, EOG was obtained from electrodes placed on the temples, which would not be feasible in real-life applications, where the sensors should preferably be embedded in the earmold. Furthermore, the experimental setup was restricted by head fixation using a chin rest, such that the EOG signals did not include the effects of head movements which would be present in more natural situations.

If the *absolute* eye gaze could be estimated from sensors placed in and around the ear, this would in fact be valuable for HA steering. However, a clear understanding of the relation between EOG and head movement is crucial for estimating the absolute eye gaze, since EOG is linked to eye gaze relative to the head. In the present study, the quality of “EarEOG” reflecting electrical signals similar to EOG but captured in the ear was investigated and the contributions of head- and eye-gaze components to it were explored. A prototype HA was designed with embedded dry electrodes in the earmolds to record the EarEOG signals (Kappel and Kidmose, 2015, 2018; Kappel et al., 2019). Furthermore, to enable motion estimation (Kok, 2016), the prototype HA included inertial sensors (i.e., accelerometer and gyroscope) and a magnetometer, referred to as magnetic inertial measurement unit (“Mag-IMU”), embedded in the behind-the-ear (BTE) shells.

In an experimental set-up, HI listeners with EarEOG electrodes and Mag-IMU sensors were placed in front of three speakers. The three speakers played sentences simultaneously and the listeners’ task was to visually focus on one of the three speakers at a time.

The EarEOG signals were modeled using the individual listeners’ saccades, eye fixations, the head rotation, and a Direct Current (DC) component (drift) commonly represented in EOG. The absolute eye gaze was estimated from the model and the Mag-IMU data. This study is the first investigation exploring the feasibility to estimate absolute eye gaze in a prototype HA with sensors placed around the ears of HI listeners.

## Materials and Methods

### Participants

Eleven HI native Danish speakers (five males, six females, aged 73 ± 4.5 years) participated in the study. Their audiograms showed a symmetric mild-to-moderate sensorineural symmetrical hearing loss. The maximum difference between the left and right ears’ audiometric thresholds (averaged between 125 and 8000 Hz) was below 10 dB, and the thresholds at 500, 1000, 2000, and 4000 Hz ranged from 36 to 56 dB HL, with an average of 45 dB hearing level (HL). All participants had normal vision or corrected vision by glasses or contact lenses.

The study was approved by The Ethics Committee for the Capital Region of Denmark (Journal number H-1- 2011-033). The study was conducted according to the Declaration of Helsinki, and all participants signed a written consent prior to the experiment.

### Experimental Setup

**FIGURE 1:**
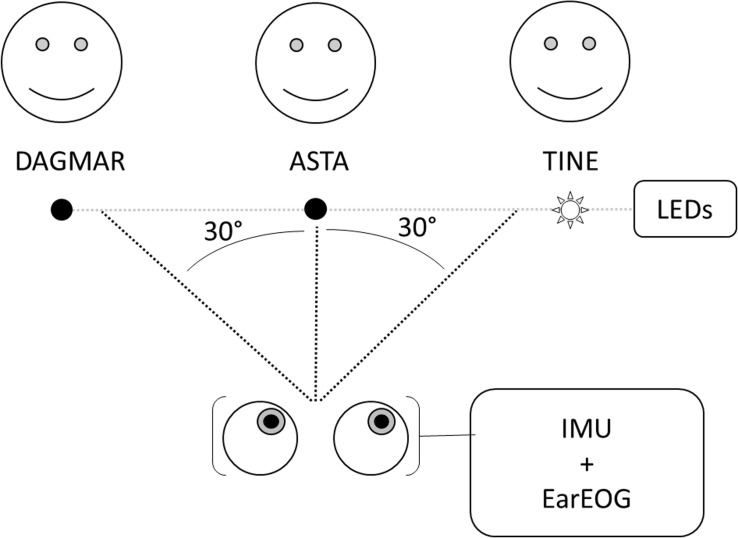
Representation of the experimental setup. The position of Dagmar, Asta and Tine were randomized between participants.

The participants were seated in a listening booth with controlled light conditions and three loudspeakers positioned at −30, 0, and +30 degrees azimuth (see Figure 1). The participants were presented speech-on-speech material from the Danish DAT speech corpus (Bo Nielsen et al., 2014), with different sentences being spoken simultaneously by three female speakers. The sentences of DAT are in the form of “Dagmar/Asta/Tine taenkte på en skjorte og en mus i går” (“Dagmar/Asta/Tine thought of a shirt and a mouse yesterday”). *Skjorte (shirt)* and *mus (mouse)* are two target words that change from sentence to sentence and between each talker. A light-emitting-diode (LED) was placed next to each of the three loudspeakers to indicate on which talker the participant should be focusing during the presentation of the sentences and they were instructed to repeat the two target words said by that talker. The LED was switched on 1 s before the beginning of the sentence and remained activated until the following target change. The participants were aided with a HA prototype that took their audiogram into account and their task was to repeat the two target words in the sentence of the target speaker at the end of the given sentence. The data was evaluated in terms of correct speech intelligibility, however, these results are not reported here since they were not the focus of the present investigation.

Two conditions were presented to the participants in blocks of six lists of twenty sentences. In the “head-fixed” condition, the participants’ head was fixed on a chin-rest to validate the eye-gaze pattern estimated from the EarEOG. In the “head-free” condition, the participants were free to move their head naturally. The head-free condition was used to investigate the interaction between the eye-gaze pattern estimated from EarEOG and the head movements estimated by Mag-IMU. The order of the conditions was randomized across participants. Each target was presented at least six times during a list of twenty sentences, and each of the nine possible transitions between two targets (from left to middle, left to right, middle to right etc., as well as the no-transition configurations where that the target remained at the same position) occurred at least twice. Before the presentation of each list, the participants were asked to fixate their eyes at the middle target. The last two transitions were chosen randomly. The participants were instructed to look at the loudspeaker indicated by the activated LED.

### Hardware

#### EarEOG Recordings

**FIGURE 2:**
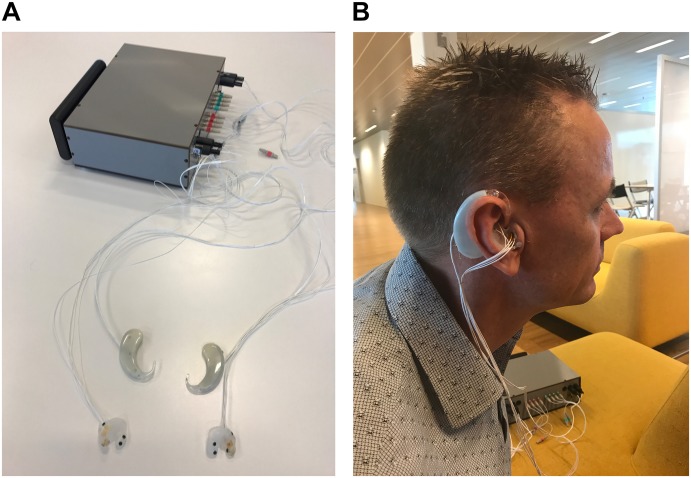
**(A)** Equipment used to record the eye gaze and head tracking signals. The earmolds contain the dry EarEOG sensors, and were individually fitted to each participant. The behind-the-ear (BTE) shells contained the Mag-IMU. The gray box is the processing unit of the prototype hearing aid, which amplifies and process all the signals from the EarEOG and motion sensors. Additionally, the gray box takes a trigger signal as input, to align the sensor signals to the presentation of visual (LED) and auditory (speech) stimuli. **(B)** Position of the hardware components during the experiment. The dry EarEOG sensors were placed in the ear canal and in the concha. The Mag-IMU were placed in the BTE shells. The gray box with the amplifier and processing unit was placed on a stable surface next to the hearing-impaired participant with cables connecting the sensors to the box. Written informed consent was obtained from the individual present in this picture for the publication of this image.

The platform used to record EarEOG signals in this study has been described previously (Kappel et al., 2019). The dry contact electrodes are based on a titanium (Ti) substrate coated with iridium-oxide (IrO2) and mechanically designed to be embedded into a soft-earpiece. The amplifier used in this setup was designed for low power and low voltage, which resulted in a small dynamic range in the order of 1 mV. A DC cancelation feedback loop maintained the signal within the dynamic range of the amplifier. To obtain an optimal fit of the electrodes, the soft silicone earmolds for each participant, depicted in Figure 2, were obtained from individual ear impressions. Six dry electrodes were positioned in each of the earmolds and placed in the participant’s ear canal and concha (Kappel et al., 2019). Each electrode was tested individually in order to ensure the best signal quality. During this test, two additional electrodes were placed near the participant’s eyes with good contact between the electrode and the skin. Those two electrodes were used as ground and reference, respectively, to evaluate the quality of the signals from each of the in-ear electrodes; they were discarded during the actual experiment. By visual inspection, the EarEOG signals with the highest signal-to-noise ratio (SNR) in the periods of eye-gaze changes were selected for further analysis.

The SNR was estimated by comparing the amplitude of the saccades to the amplitude of the noise present in the signal when the eyes were fixated. Not all electrodes provided a “good” signal; therefore the EarEOG signal studied in this experiment consisted of the best electrode in the right ear referenced to the best electrode in the left ear, sampled at 1000 Hz. When the electrode setup was established, prior to data collection, the participants were asked to move their eyes from left to right to evaluate if the correct EarEOG signal was detected. The EarEOG signals were transmitted by shielded cables to the gray box containing the amplifiers as illustrated in Figure 2. Thus, the raw signals were transmitted to the system without any pre-amplification at the ear site.

#### Head-Tracking Recordings

To estimate the head orientation, inertia sensors consisting of an accelerometer and a gyroscope as well as a magnetometer were embedded in the behind-the-ear (BTE) shell (left side). The motion sensors were based on InvenSense ICM-20948 devices. Signals were recorded at a sampling frequency of 102.27 Hz. To minimize the influence of errors due to perturbations of the magnetic field around the participant, the calibration of the magnetometer was performed by utilizing non-linear least square estimation (Kok et al., 2012). The perturbations were assumed to be constant during the experiment and were used to correct measurements from the magnetometer. To monitor those perturbations, the participant rotated the head horizontally from left to right for 10 s, while wearing the BTE shell.

### Data Analysis

#### EarEOG Signal Processing

Eye gaze was estimated from the EarEOG signals in the segments from 1 s before a switch of the LED position to 3.5 s after. Thus, the segments consisted of 1 s related to the previous gaze estimation, a short transition where the LED changed its position, and about 3 s of steady-state eye-gaze during speech presentation. The signal was low-pass filtered with a cut-off frequency of 10 Hz, after which the mean was removed. Traces were categorized according to each of the nine possible transitions between two targets. All trials were included in the analysis without artifact rejection. The EarEOG signals were recorded for both the head-fixed and the head-free condition. In the latter condition, the traces were compared to the head rotation angles estimated from the Mag-IMU. For a single participant in the head-fixed condition, the recordings in response to three sentences were removed due to trigger issues.

#### Mag-IMU Processing

The head rotation angle was estimated by applying an Extended Kalman Filter (EKF) (Kok, 2016) to the signals from the Mag-IMU. The EKF is applied in a two-step procedure, including a time update and a measurement update. The time update was estimated based on the integration of the previous gyroscope data and the measurement update was calculated from the current accelerometer and magnetometer data.

#### Modeling EarEOG to Estimate Absolute Eye Gaze

**FIGURE 3:**
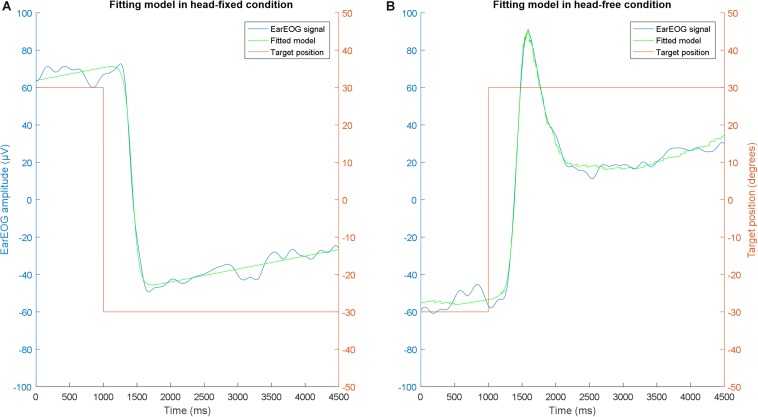
Example of the shape of the EarEOG model in the head fixed condition in one transition **(A)**, and example of the shape of the EarEOG model in the head free condition and of the estimated head rotation angle for one participant for one transition **(B)**. Both are displayed along with the target indicated to the participant.

It was assumed that the absolute eye gaze switches rapidly from one target to another, following a saccade, and then remains fixated on the new target. Curve fitting was applied to the EarEOG data using a trust-region-reflective least squares algorithm (Mord and Sorensen, 1983) with fixed boundaries for the different parameters. The shape of the curve for a saccade followed by a fixation was assumed to be similar to a hyperbolic tangent, following (Hládek et al., 2018). Head movements were estimated using the Mag-IMU data. EarEOG typically includes a DC drift in the signal due to the interface between the electrode and the skin (Huigen et al., 2002). This DC drift can be described by a linear function. As a result, an approximation of the EarEOG signal, termed “EarEOG_model_”, in μV, can be described as:

(1) EarEOG(t)model=A∗tanh[B∗(t-C)]-D∗headangle(t)+E∗t+F

with A representing the amplitude of the saccade, B the speed of the transition, and C indicating the reaction time of the participant. D reflects the ratio of degrees per μV for the EarEOG, indicating how much the eyes move when a change of 1 μV is measured in the EarEOG. E and F are parameters to describe the drift. This model does not take noise into account. An example of the model fitted to actual data for the fixed-head condition is shown in Figure 3A, this data shows a typical saccade added to a DC drift. A corresponding function for the head-free condition is shown in Figure 3B, this data indicates at first a fast eye-movement saccade, followed by a slower head movement with compensatory antiphasic eye-movements while assuming that the absolute eye-gaze is fixated on the target. The fitting algorithm was applied to the data in the interval between 1 s before the LED change and 3.5 s after the LED change.

The parts in the model that relate to the head movement [D × head angle(t)] and to the drift (E × t + F) can be separated from the absolute eye-gaze. Additionally, because of the drift, the EarEOG does not provide information about the current eye-gaze position. Therefore, removing the drift and the head movement and adding the previous eye-gaze position to the measured EarEOG signal should provide the absolute eye-gaze.

The absolute eye gaze, in degrees, estimated from the actual recorded EarEOG data, termed “EarEOG_data_”, following a transition is described as:

(2) EyeGaze(t)=k∗(EarEOG(t)data+D∗headangle(t)-E∗t-F)+prevtarget

where K is the ratio of μV to degrees, estimated by assuming that the eyes of the participants switch between the three targets over time; and “prev target” is the position of the previous target in degrees.

### Statistical Analysis

Descriptive statistics are reported as the mean (±SD) unless otherwise indicated. *T*-tests were carried out to compare the amplitude of the saccade estimated by the fitting algorithm between the nine possible transitions, for the head-fixed condition and the head-free condition. The *p*-values were Bonferroni corrected (Cleophas and Zwinderman, 2016), i.e., they were multiplied by 36, the number of possible combinations between two transitions. After these corrections, *p* < 0.05 was considered statistically significant. The validity of the models was evaluated using the adjusted coefficient of determination *R*^2^. All statistical analysis was performed with the MATLAB R2016a software (Mathworks Inc., Natick, MA, United States).

## Results

### Head-Fixed Condition

**FIGURE 4:**
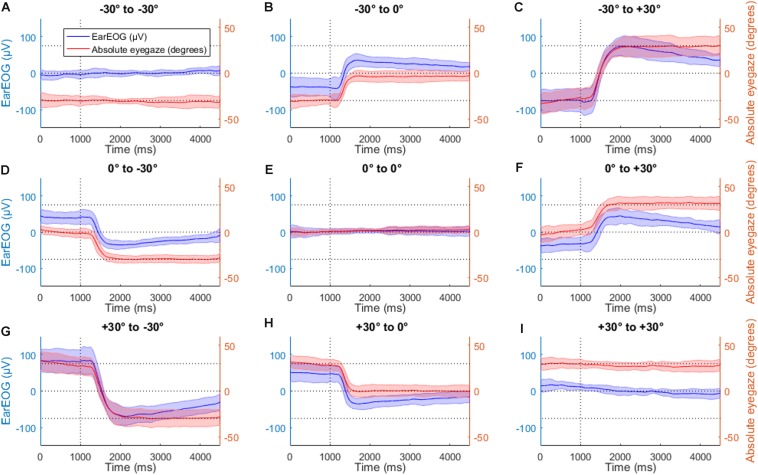
The mean EarEOG traces for all nine possible transitions **(A–I)**, in the head fixed condition, arranged in three rows and three columns are represented in blue (left axis), with the standard deviation represented as the shaded area. The row indicates the original target position (from top to bottom: left target, middle target and right target) and the column indicates the new target after the transition (from left to right: left target, middle target, right target). In the same figures, for the same transitions and the same condition, the mean absolute eye gaze is also represented in red (right axis), with the standard deviation represented as the shaded area.

Figure 4 shows the mean EarEOG traces and the mean estimated absolute eye-gaze for all nine possible transitions in the head-fixed condition, arranged in three rows and three columns. Each row indicates the original target position (from top to bottom: left target, middle target, and right target) and each column indicates the new target after the transition (from left to right: left target, middle target, right target). In the transitions where the target changes (Figures 4B–D,F–H), a few hundred ms after the LED signal (indicating a target change), a saccade can be detected. The variation of the EOG signal indicating a saccade reflects the direction of the gaze shift; an increase indicates a gaze shift to the right and a decrease indicates a gaze shift to the left. The amplitude of the response indicates the angle of the gaze shift, i.e., the higher its absolute value the larger is the gaze shift). In the head-fixed condition, the mean estimated absolute eye-gaze is positioned closely to the attended target while the sentence is playing.

**FIGURE 5:**
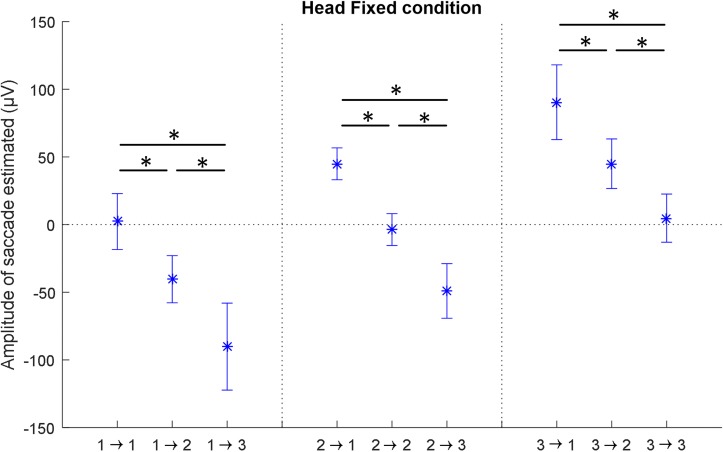
The mean values for the amplitude of the saccades for each possible transition, with the standard deviation between participants, estimated by fitting the model to the EarEOG data, in the head-fixed condition. There are significant differences between all the transitions with different angle, indicated by a ^∗^ (*p* < 0.05).

In order to explore the robustness of the EarEOG signals, the saccades were quantified for each subject as parameter A when fitting the model described in Eq. 1, the corresponding values are presented in Figure 5. The average adjusted coefficient of determination of the model fitting for this condition is 90.5% (±4.9%). The amplitude estimated for all the transitions starting from the target 1 are significantly different from each other, i.e., transition “−30° to −30°”, transition “−30° to 0°” and transition “−30° to +30°” are all significantly different. The same applies for the transitions starting from the target 2 and the transitions starting from the target 3. Therefore, knowing the previous target makes it possible to statistically discriminate between the transitions.

It can be noted that the estimated amplitude of the saccade is consistent with the angle between the two targets during a transition, i.e., the amplitude of a saccade for a 30-degree transition will be roughly half the amplitude of a 60-degree transition. Furthermore, there are no significant differences were found between the estimated parameter for transitions of the same amplitude, i.e., between transitions “−30° to 0°” and “0° to +30°” representing transitions of +30 degrees, between transitions “0° to −30°” and “+30° to 0°” representing transitions of −30 degrees and between transitions “−30° to −30°”, “0° to 0°” and “+30° to +30°” representing transitions of 0°.

### Head-Free Condition

**FIGURE 6:**
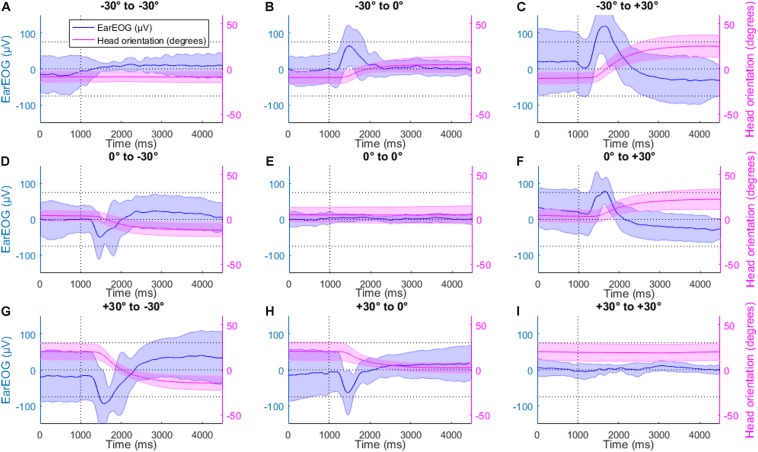
The mean EarEOG traces for all nine possible transitions **(A–I)**, in the head-free condition, arranged in three rows and three columns are represented in blue (left axis), with the standard deviation represented as the shaded area. The row indicates the original target position (from top to bottom: left target, middle target and right target) and the column indicates the new target after the transition (from left to right: left target, middle target, right target). In the same figures, for the same transitions and the same condition, the mean head rotation angle is also represented in magenta (right axis), with the standard deviation represented as the shaded area.

Figure 6 shows the corresponding mean EarEOG signals and head rotation angle estimations obtained for the head-free condition, arranged in the same way as Figure 4. In the transitions where the target changes, a few hundred ms after the target change, a rapid change occurred in the EarEOG signal, similar to the saccades present in Figure 4, followed by a slower opposite pattern bringing the signal back to approximately its original value. That slower transition appears to mirror the head movement present on the same figure. In this condition, the mean EarEOG signal is more complex and not similar to the anticipated absolute eye-gaze that the participants would have in this condition.

**FIGURE 7:**
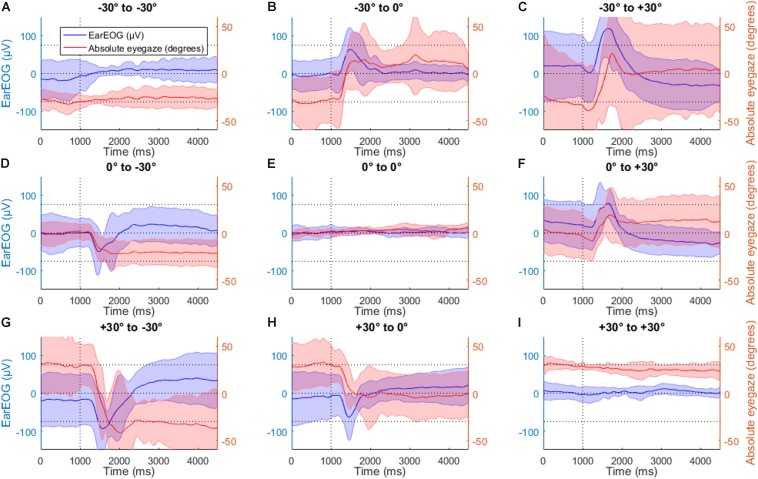
The mean EarEOG traces for all nine possible transitions **(A–I)**, in the head-free condition, arranged in three rows and three columns are represented in blue (left axis), with the standard deviation represented as the shaded area. The row indicates the original target position (from top to bottom: left target, middle target and right target) and the column indicates the new target after the transition (from left to right: left target, middle target, right target). In the same figures, for the same transitions and the same condition, the mean absolute eye-gaze is also represented in red (right axis), with the standard deviation represented as the shaded area.

Figure 7 shows the same EarEOG signals with the corresponding estimated absolute eye gaze. In the panels indicating target changes (Figures 7D,G,H), the mean of the absolute eye gaze is close to the attended target, however, the variance of the signal is too large to allow a reliable selection of the correct target. In the other panels where the target changes (Figures 7B,C,F), the mean of the absolute eye gaze does not reach the attended target, and the variance is again too large to trust the estimated value.

**FIGURE 8:**
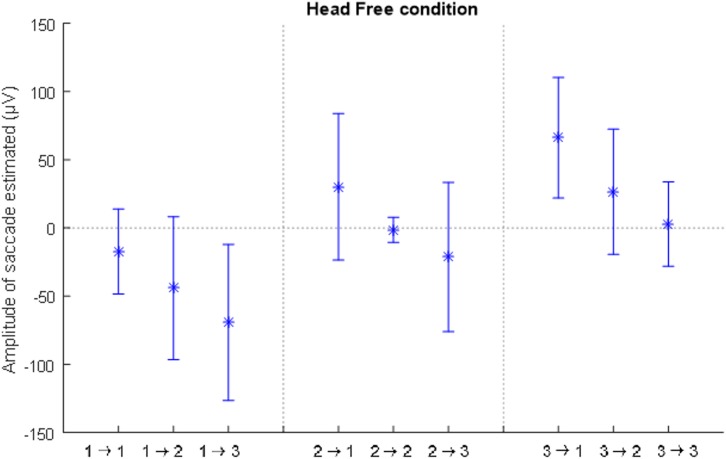
The mean values for the amplitude of the saccades for each possible transition **(A–I)**, with the standard deviation between participants, estimated by fitting the model to the EarEOG data, in the head-free condition. There are no significant differences between the transitions.

The average adjusted coefficient of determination of the model fitting for this condition is 82.6% (±8.2%). Figure 8 shows the estimated coefficient representing the amplitudes of the saccade per transition in the head-free condition, which would correspond to the parameter A from Eq. 1. Although the amplitude of the fitted saccade follows a trend similar to the saccade amplitude in the head-fixed condition, the difference between the transitions is not significant.

**FIGURE 9:**
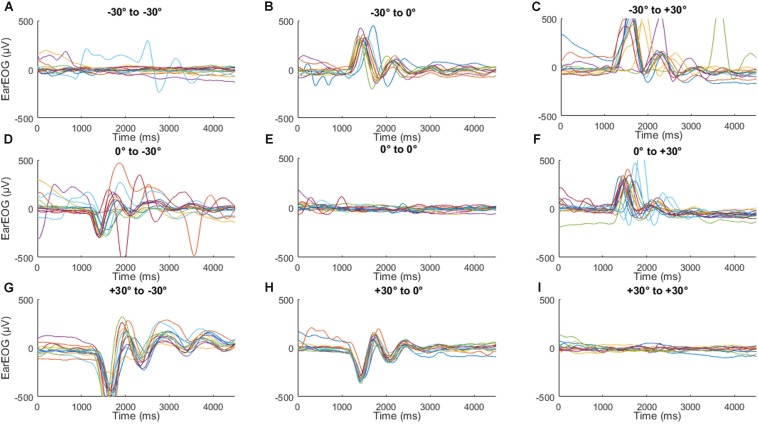
Traces of EarEOG of each trial for one participant **(A–I)**, organized following the same order for transitions as in previous figures to highlight some oscillating noise that appears when that participant moves their head.

In order to understand why the variance was so much wider in the head-free condition, the individual traces for the participants were examined. Figure 9 shows individual traces of EarEOG for one of the participants. Similar signals were present for three of the participants. It can be seen that for the transitions where the target position changed, i.e., when the participants move their head, the EarEOG seems to follow a dampened oscillation, which differs from the general trend in the mean EarEOG data. The amplitude of these oscillations is also larger than the average EarEOG signal shown in Figures 6, 7. However, those oscillations are not random since they are present and repeated consistently in most of the trials where the participants moved their head. Those oscillations could probably be explained by cable artifacts.

## Discussion

In this study, dry electrodes positioned in the ear were used to estimate EOG and a Mag-IMU was used to estimate head movement in a dynamic multi-talker situation. The EarEOG signal was modeled using saccades, head movement, and a drift. When the head was fixed, the signal from the in-ear electrodes followed patterns that are typical for EOG, representing saccades and fixations. On average, the model accounted for 90.5% of the variance of the signals averaged over all trials for a participant. Therefore, the model described here appears to be a good approximation of the signal recorded from in-ear electrodes in situations where the participant switches visual attention between a few targets. This model led to an estimation of the absolute eye gaze of the user. The analysis of the amplitudes of the saccades fitted to the mean EarEOG for each transition in the head-fixed condition showed that it was possible to statistically discriminate between the saccades depending on the angle switch followed by the eyes of the participant and therefore estimate the new target if the previous target is known.

In contrast, when the head was free to move, the signal was more variable and challenging to interpret. On average, the model still explained 82.6% of the variance of the signals, averaged over all trials for a participant. A substantial amount of the uncertainty was found when the head was free to move; furthermore, the amplitude of the saccades was not found to be statistically different between the transitions.

The device used in the present study could have recorded up to four EarEOG signals. However, although the electrode selection procedure attempted to achieve the best signals possible, it was lengthy, and a good quality EarEOG signal could not always be obtained. Therefore, in this study, only one EarEOG signal was utilized for each participant. The combination of several high quality EarEOG signals would reduce the amount of noise in the signal and highlight the eye movement features, like saccades and the vestibulo-ocular reflex.

The model parameters were estimated based on averaged data over at least 12 trials for each transition for each participant. The fitting of that model would not be as robust if it were applied to single trials of the data since those signals would be noisier and the model does not account for noise.

Long cables were used between the electrodes in the participant’s ears and the box where the amplifier was located (see Figure 2), and no pre-amplifier near the electrodes was used. Further investigations showed oscillations, such as those displayed in Figure 9, when cables were moved intentionally, which induced currents in the cables that drove the amplifier out of range. A cutoff of a few mHz instead of 0.5 Hz in the amplifier made the DC-feedback loop extremely slow, i.e., high settling time. This resulted in an artifact from the long cables when the head was turned due to the dynamics of the DC-loop seen as those oscillations in the EarEOG signal. This might have introduced other variances in the signals that the present model does not account for. Further studies on this should include pre-amplifiers near the ear to avoid the presentation of such errors.

The analysis of the model was not provided in real time, and thus, the estimation of the absolute eye gaze here was not in real-time. This would be needed to be able to steer a hearing aid via eye-gaze. The head rotation angle was estimated in real time, fitting a saccade can also be done in real time (Hládek et al., 2018) and a Kalman filter could be used to estimate the drift (Roth and Gustafsson, 2011). Thus, eye gaze can, in principle, be estimated in real-time using Mag-IMU close to the ear and dry EarEEG electrodes with pre-amplifiers near the ear.

Evidently, a system that would only ever amplify sound from positions the users’ head is pointing at might be problematic, as it would prevent the user from moving their eyes freely. Therefore, additional processing would be required to anticipate the user’s need for controlling the steering with the eyes. To apply those real-time estimations of eye-gaze in audio steering, it would be insightful to have more information about the behavior of hearing-aid users. Further studies might investigate how HI people behave in multi-talker situations using ideal motion tracking and eye-tracking devices. It would also be valuable to study how people move their eyes and their head in a situation where the audio signals could be controlled with an ideal eye-gaze detection system.

## Conclusion

Electrooculography in the ear signals were modeled in a dynamic competing talker scenario using saccades, fixations, head movements and drift. This model accounted for more than 80% of the variance of the EarEOG signals despite some hardware limitations that created additional noise. Once those technical issues are solved, the absolute eye-gaze should be able to be estimated using EarEOG and Mag-IMU sensors.

## Data Availability Statement

The datasets generated for this study are available on request to the corresponding author.

## Ethics Statement

The studies involving human participants were reviewed and approved by The Ethics Committee for the Capital Region of Denmark (Journal number H-1- 2011-033). The patients/participants provided their written informed consent to participate in this study.

## Author Contributions

All authors contributed to the study concept and design and critical revision of the manuscript for important intellectual content. AF-F, TB, MS, and SR-G acquired the data. AF-F analyzed the data. AF-F, CG, TL, and TD interpreted the results. AF-F drafted the manuscript.

## Conflict of Interest

AF-F, CG, TB, MS, SR-G, and TL are employed by Eriksholm Research Centre – part of Oticon. MR is employed by UNEEG Medical A/S. The remaining author declares that the research was conducted in the absence of any commercial or financial relationships that could be construed as a potential conflict of interest.

## References

[B1] Arons B. (2000). A review of the cocktail party effect. The separation of speech channels early work. *Transition* 16 1–2.

[B2] Bee M. A. Micheyl C. (2008). The “Cocktail Party Problem”: What Is It? How Can It Be Solved? And Why Should Animal Behaviorists Study It? *J. Comp. Pshychol.* 122 235–251. 10.1037/0735-7036.122.3.235 18729652 PMC2692487

[B3] Bo Nielsen J. Dau T. Neher T. (2014). A Danish open-set speech corpus for competing-speech studies. *J. Acoust. Soc. Am.* 135 407–420. 10.1121/1.4835935 24437781

[B4] Cleophas T. J. Zwinderman A. H. (2016). *Clinical Data Analysis on a Pocket Calculator.* Berlin: Springer International Publishing, 99–102. 10.1007/978-3-319-27104-0

[B5] Dau T. Maercher Roersted J. Fuglsang S. Hjortkjær J. (2018). Towards cognitive control of hearing instruments using EEG measures of selective attention. *J. Acoust. Soc. Am.* 143:1744 10.1121/1.5035691

[B6] Favre-Félix A. Graversen C. Hietkamp R. K. Dau T. Lunner T. (2018). Improving Speech Intelligibility by Hearing Aid Eye-Gaze Steering: Conditions With Head Fixated in a Multitalker Environment. *Trends Hear.* 22 1–13.

[B7] Fuglsang S. A. Dau T. Hjortkjær J. (2017). Noise-robust cortical tracking of attended speech in real-world acoustic scenes. *Neuroimage* 156 435–444. 10.1016/j.neuroimage.2017.04.026 28412441

[B8] Hládek Ĺ Porr B. Owen Brimijoin W. (2018). Real-time estimation of horizontal gaze angle by saccade integration using in-ear electrooculography. *PLoS One* 13:e0190420. 10.1371/journal.pone.0190420 29304120 PMC5755791

[B9] Huigen E. Peper A. Grimbergen C. A. (2002). Investigation into the origin of the noise of surface electrodes. *Med. Biol. Eng. Comput.* 40 332–338. 10.1007/BF02344216 12195981

[B10] Kappel S. L. Kidmose P. (2015). Study of impedance spectra for dry and wet EarEEG electrodes. *Proc. Annu. Int. Conf. IEEE Eng. Med. Biol. Soc.* 2015 3161–3164. 10.1109/EMBC.2015.7319063 26736963

[B11] Kappel S. L. Kidmose P. (2018). Real-Life Dry-Contact Ear-EEG. *Proc. Annu. Int. Conf. IEEE Eng. Med. Biol. Soc.* 2018 5470–5474. 10.1109/EMBC.2018.8513532 30441575

[B12] Kappel S. L. Rank M. L. Toft H. O. Andersen M. Kidmose P. (2019). Dry-Contact Electrode Ear-EEG. *IEEE Trans. Biomed. Eng.* 66 150–158. 10.1109/TBME.2018.2835778 29993415

[B13] Kok M. (2016). *Probabilistic Modeling for Sensor Fusion With Inertial Measurements*, 97–103. Available at: http://theses.eurasip.org/theses/699/probabilistic-modeling-for-sensor-fusion-with/

[B14] Kok M. Hol J. D. Schon T. B. Gustafsson F. Luinge H. (2012). “Calibration of a magnetometer in combination with inertial sensors,” in *Proceedings of the 15th International Conference on Information Fusion*, (Singapore), 787–793.

[B15] Mesgarani N. Chang E. F. (2012). Selective cortical representation of attended speaker in multi- talker speech perception. *Nature* 485 233–236. 10.1038/nature11020 22522927 PMC3870007

[B16] Mord J. J. Sorensen D. C. (1983). Computing A Trust Region Step. *SIAM J. Sci. Stat. Comput.* 4 553–572. 10.1137/0904038

[B17] O’Sullivan J. A. Power A. J. Mesgarani N. Rajaram S. Foxe J. J. Shinn-Cunningham B. G. (2015). Attentional Selection in a Cocktail Party Environment Can Be Decoded from Single-Trial EEG. *Cereb. Cortex.* 25 1697–1706. 10.1093/cercor/bht355 24429136 PMC4481604

[B18] Pomper U. Chait M. (2017). The impact of visual gaze direction on auditory object tracking. *Sci. Rep.* 7 1–16. 10.1038/s41598-017-04475-1 28680049 PMC5498632

[B19] Puder H. (2009). “Hearing Aids: An Overview of the State-of-the-Art, Challenges, and Future Trends of an Interesting Audio Signal Processing Application,” in *Proceedings of the 6th International Symposium on Image and Signal Processing and Analysis*, (Piscataway, NJ: IEEE), 1–6.

[B20] Roth M. Gustafsson F. (2011). “An efficient implementation of the second order extended Kalman filter,” in *Proceedings of the 14th International Conference on Information Fusion (Fusion 2011)*, (Chicago, IL), 1–6.

